# Validation of a brief version of the Difficulties in Emotion Regulation Scale (DERS-16) with an older Norwegian population

**DOI:** 10.1007/s10433-023-00775-w

**Published:** 2023-06-22

**Authors:** Endre Visted, O. A. Solbakken, S. Mæland, L. T. Fadnes, L. B. Bjerrum, I. H. Nordhus, E. Flo-Groeneboom

**Affiliations:** 1 Department of Clinical Psychology, University of Bergen, Årstadveien 17, 5009 Bergen, Norway; 2 Department of Psychology, University of Oslo, P.O Box 1094, Blindern, 0317 Oslo, Norway; 3 Department of Global Public Health and Primary Care, University of Bergen, P.O Box 7804, 5020 Bergen, Norway; 4 Bergen Addiction Research, Department of Addiction Medicine, Haukeland University Hospital, 5021 Bergen, Norway

**Keywords:** Emotion regulation, Validation, Psychometric properties, DERS-16

## Abstract

Emotion regulation is proposed to have a salient role in optimal aging. However, currently used measures of emotion regulation have not been validated for older adults. Therefore, we evaluated the psychometric properties of the Difficulties in Emotion Regulation Scale—short form (DERS-16) in a large Norwegian sample consisting of individuals between 70 and 95 years (n = 2525). Tests of internal consistency, reliability, assessment of intra-domain correlations, and confirmatory factor analyses were performed. Construct validity was further investigated by assessing concurrent associations between DERS-16 and well-established measures of psychological disorders, psychological health, and well-being (five-item version of Geriatric Depression Scale, Geriatric Anxiety Inventory—short form, and OECD guidelines on measuring subjective well-being). All subscales derived from the instrument showed adequate internal consistency. Furthermore, we obtained a theoretically consistent factor structure, in which a bifactor model combining a general emotion regulation factor and five additional domain-specific facet-factors had superior model fit. As expected, difficulties in emotion regulation correlated positively with symptoms of depression and anxiety, and negatively with psychological health and well-being. Associations were generally of moderate strength. We can thus conclude that the DERS-16 demonstrates excellent psychometric properties when used in samples with older adults and may safely be employed in studies of emotion regulation difficulties in the older segment of our population.

**Supplementary Information:**

The online version contains supplementary material available at 10.1007/s10433-023-00775-w.

## Introduction

The way people relate to and regulate their emotions is associated with psychological health. A common definition of emotion regulation is “the processes by which individuals influence which emotions they have, when they have them, and how they experience and express these emotions” (Gross 1998, 2015). Empirical evidence points out emotion regulation as a general transdiagnostic factor that may explain the development and maintenance of psychological disorders (Aldao et al. 2010). Numerous self-report questionnaires have been designed to assess emotion regulation (John and Eng 2014). However, most of these questionnaires have been designed using samples with younger adults. The validity of emotion regulation assessment with older adults is therefore unclear.

One of the most frequently used measures of emotion regulation is the Difficulties in Emotion Regulation Scale (DERS-36; Gratz and Roemer (2004)). The DERS-36 is a 36-item questionnaire designed to measure emotion regulation abilities. Results from studies that explore the psychometric properties, and the factorial structure of the DERS-36 are not conclusive (Lee et al. 2016; Osborne et al. 2017). Additionally, attempts have been made to design shorter versions of the DERS-36, to reduce the administration time and the burden for research participants and patients. Subsequently, shorter forms of the DERS-36 based on improved psychometric properties have been developed. One of these is the DERS-16 (Bjureberg et al. 2016), which omits one of the original subscales (emotional awareness) and is shortened down to 16 items.

The DERS-16 measures emotion regulation difficulties across five dimensions: (1) emotional clarity and understanding; (2) acceptance of emotions; (3) engaging in goal-directed behavior when experiencing emotions; (4) refraining from impulsive behaviors when experiencing emotions, and (5) access to effective and situationally appropriate strategies to regulate emotions. Several attempts have been made to investigate the psychometric properties and factor structure using Confirmatory Factor Analyses (CFA), but the results are inconsistent. Studies have demonstrated acceptable fit when examining the factor structure of the DERS-16 using a five-factor model, both in university students (Shahabi et al. 2018; Yiğit and Guzey Yiğit 2019) and hospitalized patients with severe psychiatric disorders (Charak et al. 2019). In contrast, poor or marginal fit indices for the five-factor model was found in studies that used samples that were large and community-based (n > 700) (Miguel et al. 2017; Sörman et al., 2022), and outpatients with psychosis (Lawlor et al. 2021). Finally, two studies found satisfactory fit using a five-factor model when omitting two items from the strategy-factor in their analyses, hence testing a 14-item version of the DERS-16 (Lawlor et al. 2021; Westerlund and Santtila 2018).

Concerning other models, Shahabi et al (2018) tested a second-order model including the five factors in addition to a higher-order emotion regulation difficulties factor. However, the model showed poor fit. More recently, several studies have evaluated a bifactor solution of the DERS-16, with five factors in addition to a general emotion regulation difficulties factor. Sörman et al. (2022) found good and superior fit in comparison to the five-factor solution. Similar results have been found in studies using clinical samples with eating disorders, including adult women (Nordgren et al. 2019) and adolescent girls (Monell et al. 2022). This is in line with factor-analytic investigations with the DERS-36, where best fit is typically demonstrated using a bifactor solution in comparison to five-factor and second-order models (Hallion et al. 2018; Osborne et al. 2017).

Regarding construct validity of the DERS-16, studies show promising results, as self-reported difficulties in emotion regulation correlate with symptoms and severity of depression (Burton et al. 2022; Skutch et al. 2019) and anxiety (Shahabi et al. 2018; Sörman et al. 2022). In sum, former factor-analytic investigations show somewhat mixed results, of which heterogeneity in terms of types of statistical models and sample characteristics are common.

Another concern regarding studies based on the DERS-16, is the lack of older adults in the study samples. Although five studies included smaller sub-samples of older adults (Charak et al. 2019; Lawlor et al. 2021; Miguel et al. 2017; Sörman et al. 2022; Westerlund and Santtila 2018), the exact distribution of age groups in these studies are unclear, and most studies do not consider age as a possible contributor to the results. An exception is Miguel et al. (2017) who included a sample with individuals ranging in age from 18 to 70 years and found a negative correlation between age and DERS-16. That is, the older the individuals, the lesser self-reported emotion regulation difficulties. In line with Miguel and colleagues, Westerlund and Santtila (2018) found that younger individuals (< 40 years old) reported significantly more difficulties with emotion regulation compared with older individuals (> 40 years old). However, their study included a small sample of older adults (n = 59), raising questions about the validity of this finding.

Overall, the lack of older adults in validation studies utilizing the DERS-16 raises concerns regarding the validity of the instrument specifically for this age group. Studies suggest that age is an important moderator in terms of emotional processing. For example, laboratory investigations show that older individuals recognize negative emotions less accurate than younger adults (Mill et al. 2009). Also, older individuals choose different emotion regulation strategies than younger individuals when experiencing negative emotions. For instance, one study showed that older individuals exhibited a greater preference for choosing distraction as a strategy for regulating their emotions compared to younger adults when exposed to negative-valanced images. This preference was further linked to higher levels of well-being among the older adult sample (Scheibe et al. 2015). Moreover, studies have suggested that the experience of negative affect decreases as a function of age (Furnham and Cheng 2019). One study demonstrated that older individuals reported fewer negative emotions during an unpleasant social interaction compared to their younger counterparts. This was further supported by lower levels of psychophysiological arousal among the older group (Luong and Charles 2014). Additionally, older individuals experience less negative and more positive affect in daily life (Hay and Diehl 2011) along with more stability in emotional states (Brassen et al. 2011). Finally, older adults attend to and remember positive stimuli and exhibit less attention to negative stimuli compared to younger adults (Reed et al. 2014). Based on such findings, it has been hypothesized that emotion regulation difficulties decrease as an effect of age, partly because of changes in personal goals when time left in life is perceived as limited (Carstensen et al. 1999). In support of this hypothesis, studies with heterogeneous age groups report that self-reported emotion regulation difficulties as measured by the DERS-36 decreases with age (Giromini et al. 2017; Orgeta 2009). Against this backdrop, it appears imperative to test whether the assessments of self-reported emotion regulation that we use in clinical and empirical work are valid also in older populations.

The aim of the present study was to examine the internal structure of scores from the DERS-16 by analyses of internal consistency and confirmatory factor analyses (CFA) in a sample of individuals who were 70 years or older, and testing four factor-analytic models: (1) a unidimensional model based on the assumption of a general emotion regulation difficulties factor; (2) a five-factor model; (3) a hierarchical model and (4) a bi-factorial model consisting of a general emotion regulation difficulties factor together with all five factors was assessed. Based on prior studies, we hypothesized that the single-factor solution would have the poorest fit, while the proposed factor models with five distinct emotion regulation facets and the higher-order model would be confirmed with acceptable fit indices. Based on prior results from studies with the DERS-36 (Hallion et al. 2018; Osborne et al. 2017) and DERS-16 (Monell et al. 2022; Nordgren et al. 2019; Sörman et al. 2022), we predicted that the bifactor solution would demonstrate superior fit compared to the other models.

The second aim of the study was to examine construct validity of the DERS-16 with relevant external correlates. For example, difficulties in self-reported emotion regulation has been shown to be associated with major depressive disorder (Joormann and Stanton 2016; Visted et al. 2018) and anxiety disorders (Cisler and Olatunji 2012). Similarly, other studies have found positive correlations between symptoms of depression and anxiety and the DERS-16 (Bjureberg et al. 2016; Shahabi et al. 2018; Yiğit and Guzey Yiğit 2019). However, no prior studies have investigated the construct validity of psychological health using measures validated specifically for older individuals. We hypothesized to find positive relationships between self-reported emotion regulation difficulties and measures of depression and anxiety, and negative relationships between self-reported emotion regulation difficulties and well-being.

## Method

### Participants and procedures

A representative sample of 81,170 individuals from among 224,000 adult inhabitants in the City of Bergen in Western Norway were invited in April 2020 to participate in a study surveying the impact of lockdown on daily life and health during the COVID-19 pandemic. The individuals invited to participate were drawn from the Contact and Reservation Registry through the Norwegian Digitalization Agency. In total, 29,535 individuals consented to participate in the first wave of the study. Out of these, 3917 first wave responders were over 69 years old. For the present validation study, all 3917 home-dwelling seniors (age > 69 years) were invited to fill out an online questionnaire package involving psychological health and well-being.

The ethics committee Regional Ethics Committee Western Norway approved the study (Reference: 131560). The study was conducted according to the guidelines of the Declaration of Helsinki. All participants consented before participation.

### Measures

Difficulties in Emotion Regulation Scale-16 item version (DERS-16) (Bjureberg et al. 2016). The DERS-16 consist of 16 items that assess the following facets of emotion regulation difficulties: (1) nonacceptance of negative emotions (three items, labelled NONACCEPT); (2) inability to engage in goal-directed behaviors when experiencing negative emotions (three items, labelled GOALS); (3) difficulties controlling impulsive behaviors when experiencing negative emotions (three items, labelled IMPULSE); (4) limited access to emotion regulation strategies (five items, labelled STRATEGIES); and (5) lack of emotional clarity (two items, labelled CLARITY). The items are rated on a 5-point Likert-scale (from 1: almost never to 5: almost always). The total score (DERS-TOTAL) is derived by summing all individual item responses, and subscale scores are derived by summing up all item responses within each subscale. Higher scores reflect greater levels of emotion regulation difficulties. The DERS-36 has been translated into Norwegian (Dundas et al. 2013).

The five-item version of Geriatric Depression Scale (GDS-5) (Hoyl et al. 1999). The GDS-5 was used to measure symptoms of depression. The participants respond on a two-point rating scale (0–1; e.g. “Do you often feel helpless?” and “Do you feel pretty worthless the way you are now?”). Higher scores (maximum 5) reflect greater depressive symptoms. The GDS-5 has been translated into Norwegian, and validated for use with Norwegian participants (Eriksen et al. 2019).

The short form of the Geriatric Anxiety Inventory (GAI-SF; (Byrne and Pachana 2011) was used to measure symptoms of anxiety. It consists of five items (e.g., “I worry a lot of the time” and “I often feel nervous”), requiring a yes (agree) or no (disagree) answer. Responses are summed and higher total score reflect greater anxiety. The GAI-SF has been translated and validated to be used in Norway (Molde et al. 2017).

Subjective well-being was measured using two items from the OECD Guidelines on measuring Subjective Well-being (OECD 2013). Participants respond on a scale from 0 to 10 (0: not at all satisfied/not at all worthwhile, 10: completely satisfied/completely worthwhile) on the following two items: (1) Overall, how satisfied are you with life? (2) Overall, to what extent do you feel the things you do in your life are worthwhile? Higher scores reflect higher well-being.

### Statistical analyses

#### Preliminary analyses and reliability

Frequency and descriptive analyses on background variables, mental health, and well-being were conducted on both included and excluded participants. T-test analyses on indices of mental health and well-being were performed to assess possible disparities between included and excluded participants. Descriptive analyses were performed with means and standard deviations for each item of the DERS-16. Moreover, skewness and kurtosis, means, standard deviations and Cronbach’s alpha values for each subscale was computed. In this study we adhered to the recommended spectrum (skewness < 3 and kurtosis < 10) (Kline 2016). We calculated Pearson correlation coefficients for all subscales. Preliminary analyses and correlation coefficients were calculated using IBM STATISTICS SPSS version 28.

#### Factor analysis and model fit

Four confirmatory factor analyses (CFA) were carried out based on structural equation modelling (SEM): (1) a single-factor, unidimensional “Emotion Regulation Difficulties” solution; (2) a five-factor facet solution; (3) a hierarchical model with a higher-order general factor and five second order facet factors, and finally; (4) a bifactor model, including the five facet factors in addition to a general emotion regulation difficulties factor at the same level. The fit indices used to assess model fit were comparative fit index (CFI), Tucker Lewis Index (TLI), and Root Mean Square Error of Approximation (RMSEA). We used the recommendations of Hu and Bentler (1999) in terms of the interpretation of the fit indices: values of > 0.95 on CFI and TLI and values of < 0.06 on RMSEA was considered as acceptable fit indices. The CFA analyses were performed using IBM STATISTICS AMOS version 27.

#### Associations with external correlates

Bivariate correlations were computed between all facets of the DERS-16, and indices of depression (GDS-5), anxiety (GAI-SF), and subjective well-being (OECD) were used to assess associations with relevant external variables. To assess the explained variance of the DERS-16 in the external variables, we calculated R^2^ (*r* * *r*) for strongest obtained correlations.

## Results

### Recruitment and preliminary analyses

Of the total 3917 participants that were invited, 914 responders did not answer or did not want to participate. Of the 3003 resulting participants, 478 respondents did not complete the DERS-16 or had missing data on DERS-16 and were omitted from further analyses. Thus, 2525 were included in this study (65% response rate). See Table 1 for demographic, mental health, and well-being characteristics of the included participants. Overall, the included sample reported low levels of depression and anxiety, and relatively high levels of well-being. The characteristics of the 478 respondents that were excluded from analyses are presented in Additional file 1: Table S1. Independent samples T-tests revealed no significant differences between the excluded group and the included group in terms of anxiety (t(303) = − 1.56, *p* = 0.12) or well-being (t(369) = 1.50, *p* = 0.14). However, the excluded group had elevated depression levels compared to the included sample (t(293) = − 2.03, *p* = 0.04).

**Table 1:** Demographic and background characteristics of participants (n = 2525)

	n	%
*Gender*		
Male	1356	54
Female	1169	46
*Age*		
70–74	1271	51
75–79	750	30
80–84	309	12
> 85	176	7
*Domestic status*^†^		
Living alone	1007	42
Living with others	1336	58
*Usage of health services prior month*		
Familiy doctor	13	1
Nursing service or home care	16	1
Hospital services	8	> 1
*COVID-19 related variables*		
In quarantine prior month	141	6
COVID-19 infection prior month	63	3
*Mental health and well-being indices*		M (SD)
Depression (GDS-5)	2455	0.79 (1.02)
Anxiety (GAI-SF)	2478	0.57 (1.19)
Well-being (OECD)	2507	15.28 (3.73)

GDS-5, Five item Geriatric Depression Scale; GAI-SF, Geriatric Anxiety Inventory short form; OECD, OECD Guidelines on measuring Subjective Well-being

^†^Total n was 2373 (missing data from 152 participants)

The means and standard deviations for all items are presented in Table 2.

**Table 2:** Means and standard deviations for each item of the DERS-16

Factor	Item no	Item	M	SD
Clarity	1	I have difficulty making sense out of my feelings	1.36	0.56
	2	I am confused about how I feel	1.18	0.43
Goals	3	When I am upset, I have difficulty getting work done	1.56	0.69
	7	When I am upset, I have difficulty focusing on other things	1.61	0.72
	15	When I am upset, I have difficulty thinking about anything else	1.69	0.81
Impulse	4	When I am upset, I become out of control	1.32	0.58
	8	When I am upset, I feel out of control	1.23	0.52
	11	When I am upset, I have difficulty controlling my behaviors	1.21	0.48
Nonaccept	9	When I am upset, I feel ashamed with myself for feeling that way	1.34	0.61
	10	When I am upset, I feel like I am weak	1.27	0.55
	13	When I am upset, I become irritated with myself for feeling that way	1.57	0.73
Strategies	5	When I am upset, I believe that I will remain that way for a long time	1.17	0.54
	6	When I am upset, I believe that I’ll end up feeling very depressed	1.14	0.44
	12	When I am upset, I believe that there is nothing I can do to make myself feel better	1.26	0.57
	14	When I am upset, I start to feel very bad about myself	1.42	0.67
	16	When I am upset, my emotions feel overwhelming	1.43	0.71

### Reliability

The range of skewness of the DERS-16 was 1.3–2.5, and 2.4–7.3 for kurtosis. Internal consistency was acceptable, with Cronbach´s alpha values ranging from 0.66 to 0.79 on subscales and 0.92 for the total scale. See Table 3 for correlations and Table 4 for descriptive statistics, distribution, and internal consistency values.

**Table﻿ 3:** Person correlation coeffecients between five factors of the DERS-16

	Clarity	Goals	Impulse	Strategies	Nonaccept
Clarity	–				
Goals	0.49	–			
Impulse	0.48	0.66	–		
Strategies	0.56	0.70	0.65	–	
Nonaccept	0.51	0.52	0.52	0.65	–
Total	0.68	0.87	0.80	0.90	0.80

All Pearson correlations *p* < .001

**Table 4:** Means, distribution and internal consistency of the DERS-16

	Mean	SD	Skewness	Kurtosis	α
Clarity	2.53	0.86	2.17	7.11	0.66
Goals	4.86	1.87	1.29	2.41	0.79
Impulse	3.75	1.31	2.51	8.79	0.78
Strategies	6.44	2.18	2.34	7.31	0.79
Nonaccept	4.18	1.56	2.01	5.88	0.76
Total	21.76	6.48	1.82	4.70	0.92

α, Cronbach's alpha. The mean values of each subscale of the DERS-16 were computed by summing responses within each subscale

### Confirmatory factor analyses

First, the unidimensional model indicated poor fit (RMSEA = 0.10; CFI = 0.85; TLI = 0.83). The five-factor facet model had fair fit (RMSEA = 0.08; CFI = 0.92; TLI = 0.90). The higher-order or hierarchical model showed a similar fit to the five-factor facet model (RMSEA = 0.08; CFI = 0.91; TLI = 0.90). Finally, when assessing the five-factor facet model with the addition of a general factor (bi-factor model), we found clearly superior fit compared to the other models (RMSEA = 0.05; CFI = 0.97; TLI = 0.96). See Table 5 for fit indices for all confirmatory factor analyses.

**Table 5:** Model fit indices for different factor models of the DERS-16. RMSEA presented with 95% confidence interval

	χ^2^	*df*	*p* value	RMSEA	CFI	TLI
Single factor model (unidimentional)	2873.52	104	> 0.000	0.10	0.85	0.83
Five factor model	1587.43	94	> 0.000	0.08	0.92	0.90
Higher order model	1691.00	99	> 0.000	0.08	0.91	0.90
Bifactor model	546.10	78	> 0.000	0.05	0.97	0.96

**Fig. 1:**
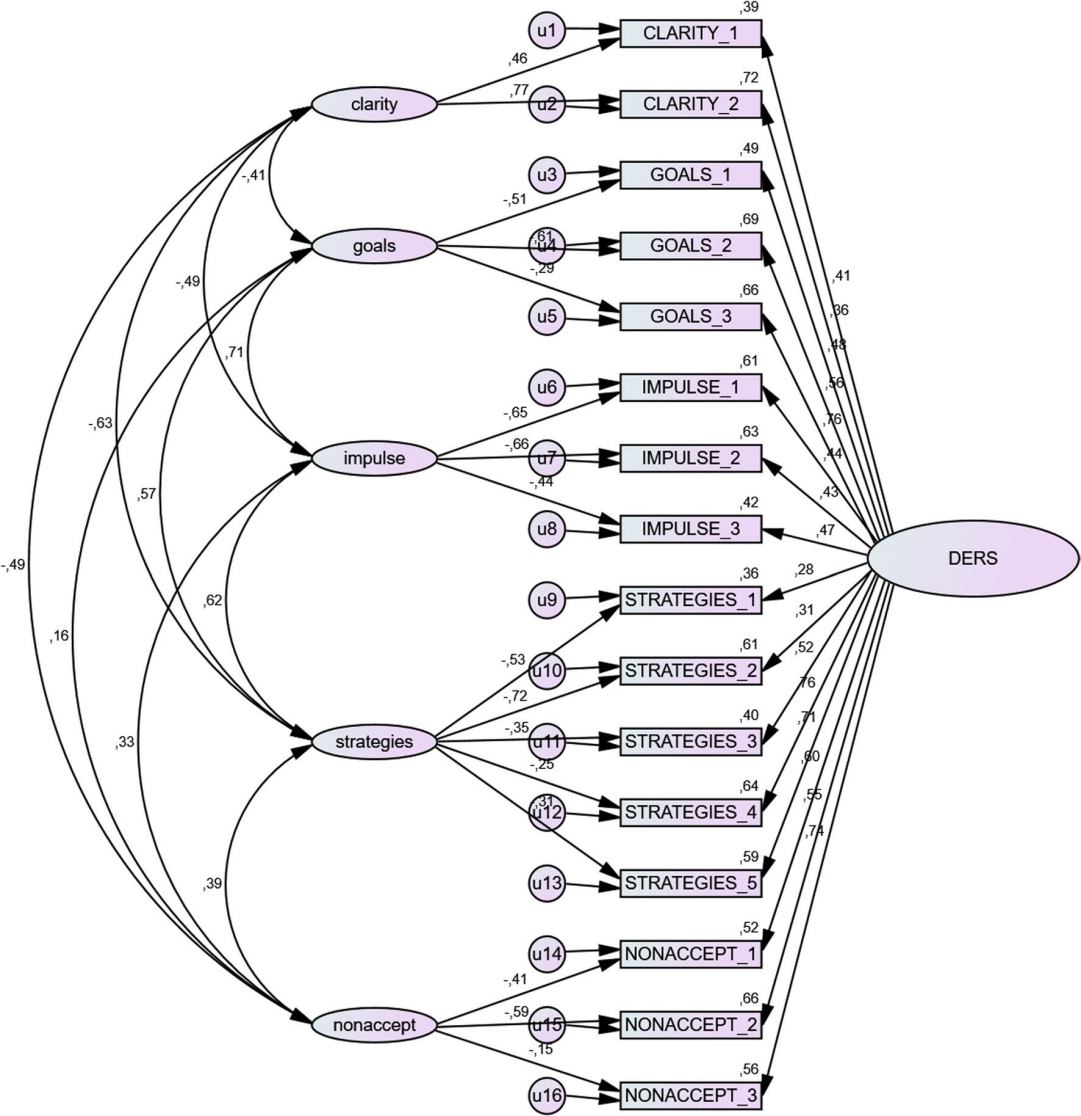
Bifactor model of DERS-16 items with standardized factor loadings

The bi-factor model is graphically represented with standardized factor loadings in Fig. 1. In this model, the five facet factors CLARITY, GOALS, IMPULSE, STRATEGIES and NONACCEPT load on every item of each factor, alongside the general emotion regulation difficulties factor (TOTAL-factor, represented in Fig. 1 as DERS).

### Associations with external correlates

The correlations between all DERS-16 scales and depression (GDS-5), anxiety (GAI-SF) and well-being (OECD) are presented in Table 6. As expected, the associations between self-reported emotion regulation difficulties, depression, and anxiety were positive and generally of moderate strength (Cohen 1988). Moreover, the associations between self-reported emotion regulation difficulties and well-being were negative, and mostly of moderate strength. The strongest correlations across the three convergent construct-variables were found for the DERS-TOTAL (explaining 14%, 24%, and 15% of the variance in depression, anxiety, and well-being, respectively) and the subscale STRATEGIES (explaining 14%, 24% and 14% of the variance in depression, anxiety, and well-being respectively).

**Table 6:** Correlations with other variables

	GDS-5	GAI-SF	OECD
DERS-total	0.37***	0.49***	− 0.39***
DERS-clarity	0.31***	0.41***	− 0.30***
DERS-goals	0.29***	0.40***	− 0.34***
DERS-impulse	0.29***	0.35***	− 0.31***
DERS-nonaccept	0.26***	0.34***	− 0.25***
DERS-strategies	0.37***	0.49***	− 0.37***

GDS-5, Five item Geriatric Depression Scale; GAI-SF, Geriatric Anxiety Inventory short form; OECD, OECD Guidelines on measuring Subjective Well-being

***p < .001

## Discussion

The DERS-16 is a promising questionnaire for measuring emotion regulation difficulties in research and clinical settings. However, no prior studies have validated the use of the DERS-16 in samples of older adults. Hence, we investigated the psychometric properties of the DERS-16 in a large sample of older individuals. Overall, our results suggest that the DERS-16 has acceptable psychometric properties when applied with older adults. Moreover, DERS-16 showed good construct validity, as difficulties with self-reported emotion regulation was substantially and positively correlated with symptoms of depression and anxiety, and equally substantially, but negatively correlated with well-being.

When considering the scores on DERS-16 of our sample, they were considerably lower (mean total score (M) = 21.76) compared to previous investigations using younger community samples, including Bjureberg et al. (2016; M = 42.90 and 33.57) Miguel et al. (2017; M = 44.57), Shahabi et al. (2018; M = 32.92), Sörman et al (2022; M = 29.13) and Yiğit and Guzey Yiğit (2019; M = 38.71). This lends support to previous findings that aging is associated with changes in emotional experience, and that increasing age is associated with decreased difficulties in emotion regulation (Giromini et al. 2017; Miguel et al. 2017; Westerlund and Santtila 2018). The lower self-reported difficulties in emotion regulation could possibly also account for the fact that older individuals typically report higher well-being and life satisfaction compared to younger adults (Carstensen et al. 2011). Taken together, our finding of lower self-reported difficulties in emotion regulation in older adults show that DERS-16 scores should be corrected for age to avoid underestimation of older individuals’ difficulties with emotion regulation (Giromini et al. 2017). This was well demonstrated in a study that included older individuals with generalized anxiety disorder that showed lower levels of self-reported emotion regulation difficulties (measured with the DERS-36) compared to younger adults who met the same diagnostic criteria (Staples and Mohlman 2012).

The analyses of skewness, kurtosis and Cronbach’s alpha showed that the DERS-16 demonstrated acceptable degrees of reliability (Kline 2016). In terms of internal consistency, all subscales except CLARITY had values over 0.75. The CLARITY subscale had an alpha of 0.66. It is likely that this is due to the fact that this scale consist of only two items, and lower number of items on a scale commonly result in lower Cronbach’s alphas (Tavakol and Dennick 2011).

The results from our confirmatory factor analyses were comparable with prior findings. In line with previous results, the five-factor model demonstrated similar fit indices (Miguel et al. 2017; Sörman et al. 2022). However, we did not replicate previous findings that showed superior fit indices for the five-factor model (Shahabi et al. 2018; Westerlund and Santtila 2018; Yiğit and Guzey Yiğit 2019). One possible explanation for the mixed results may be characteristics of the included samples across studies. The studies that found acceptable factor analytic fit indices for the five-factor solution had smaller sample sizes (n = 201 and n = 316) and recruited younger adults that were university students. The studies that did not find support for the five-factor model had larger samples (n = 725 and n = 843) and had a community sample with a wider age range. Larger and more representative samples may therefore have affected the results, which may also account for the finding in the current study. We did, in line with recent investigations find superior fit for the bifactor model (Monell et al. 2022; Nordgren et al. 2019; Sörman et al. 2022). This is also consistent with findings that used the original DERS-36 (Hallion et al. 2018; Osborne et al. 2017). The main defining feature of a good fitting bi-factor model is that it lends credence to the existence of an overarching global level factor that cuts across facet domains, while simultaneously validating any theoretically distinct lower-level facet scores. It thus mirrors the typical structure of instruments that operates with both an overall score and separate subscale scores, as do the DERS-16. Essentially, our CFAs thus indicate that both the total score of the DERS-16 and the five subscale scores are structurally valid with older individuals.

The results also support the external validity of the DERS-16, as we found significant correlations with symptoms of depression, anxiety, and well-being. This is also in line with other investigations which found similar correlation coefficients between the DERS-16 and measures of depression and anxiety (Bjureberg et al. 2016; Shahabi et al. 2018; Yiğit and Guzey Yiğit 2019).

Although the current study provided evidence for the psychometric validity of the DERS-16 in a sample of older adults, it has several limitations that need to be addressed in future research. First, the study was conducted during the COVID-19 pandemic, during which the population faced several restrictions such as reduced contact with others and demands for social distancing. However, only a small proportion of our sample was directly affected by COVID-19, either through direct infection or isolation in quarantine. Nonetheless, the prolonged threat of infection and increased isolation may have resulted in increased stress, which in turn could have affected the scores of the DERS-16. Second, as the study was conducted in Norway the generalizability of the results to other cultures may be limited. Thus, to add a comparative perspective to our study, similar investigations should be conducted across countries and cultures. Similarly, our sample reported low rates of depressive and anxiety symptoms, along with high rates of well-being. Therefore, future investigations should include older individuals within different clinical populations. Higher scores in clinical populations in comparison to healthy older individuals would further increase the validity of the DERS-16, and in addition clarify whether the clinical cut-off values should be lowered when considering self-reported emotion regulation difficulties in older individuals. Second, all measures in the current study were based on self-report questionnaires, which could be associated with biases such as social desirability and fabrication. Meanwhile, this study also has several strengths; it is based on a large sample randomly pulled from the general population of older adults, with a relatively large age span. Furthermore, the study has systematically used instruments that have been validated for use by older individuals.

In conclusion, this study was conducted with a large sample of older adults and provides evidence that DERS-16 has satisfactory psychometric properties when employed with older individuals. The DERS-16 exhibits acceptable factor-analytic fit indices and demonstrates adequate construct validity, given that the questionnaire correlates with measures of psychological health and well-being. This study indicates that age should be considered when examining emotion regulation. Further research is necessary in other cultures and among various subgroups of older individuals, including clinical populations.

## Supplementary Information

Below is the link to the electronic supplementary material.

**Additional file 1. Table 1S**: Demographic and background characteristics of participants excluded from analyses (n = 478).
