# Supplementary material for: Validation of a brief version of the Difficulties in Emotion Regulation Scale (DERS-16) with an older Norwegian population
Source: Eur J Ageing. 2023 Jun 22;20(1):26. doi: 10.1007/s10433-023-00775-w (PMC10287601; doi:10.1007/s10433-023-00775-w)
Supplement: Supplementary file 1 — Additional file 1. Table 1S: Demographic and background characteristics of participants excluded from analyses (n = 478). [file 10433_2023_775_MOESM1_ESM.docx]

| Table 1S | |  |  |  |
| --- | --- | --- | --- | --- |
| Demographic and Background Characteristics of Participants excluded from | | | | |
| Analyses (n = 478) | |  |  |  |
|  |  |  |  |  |
| Gender |  | n |  | % |
|  | Male | 220 |  | 46 |
|  | Female | 258 |  | 54 |
|  |  |  |  |  |
| Age |  | n |  | % |
|  | 70-74 | 226 |  | 47 |
|  | 75-79 | 122 |  | 26 |
|  | 80-84 | 75 |  | 16 |
|  | >85 | 50 |  | 11 |
|  |  |  |  |  |
| Domestic status | | n |  | % |
|  | Living alone | 393 |  | 82 |
|  | Living with others | 85 |  | 18 |
|  |  |  |  |  |
| Usage of health services prior month | | n |  | % |
|  | Familiy doctor | 7 |  | 1 |
|  | Nursing service or home care | 3 |  | 1 |
|  | Hospital services | 6 |  | 1 |
|  |  |  |  |  |
| COVID-19 related variables | | n |  | % |
|  | In quarantine prior month | 27 |  | 6 |
|  | COVID-19 infection prior month | 7 |  | 2 |
|  |  |  |  |  |
| Mental health and well-being indices | | n |  | M (SD) |
|  | Depression (GDS-5) | 244 |  | 0.93 (1.02) |
|  | Anxiety (GAI-SF) | 259 |  | 0.70 (1.33) |
|  | Well-being (OECD) | 306 |  | 14.92 (4.11) |
|  |  |  |  |  |
| Note. GDS-5 = Five item Geriatric Depression Scale; GAI-SF = Geriatric | | | | |
| Anxiety Inventory short form; OECD = OECD Guidelines on measuring | | | | |
| Subjective Well-being | |  |  |  |

**Supplementary materials**
